# Bypass of Booming Inputs of Urban and Sludge-Derived Microplastics in a Large Nordic Lake

**DOI:** 10.1021/acs.est.0c08443

**Published:** 2021-06-01

**Authors:** François Clayer, Morten Jartun, Nina T. Buenaventura, Jose-Luis Guerrero, Amy Lusher

**Affiliations:** † Norwegian Institute for Water Research (NIVA), Gaustadalléen 21, 0349 Oslo, Norway

**Keywords:** microplastics, lake sediment, catchment, land use, plastic sources

## Abstract

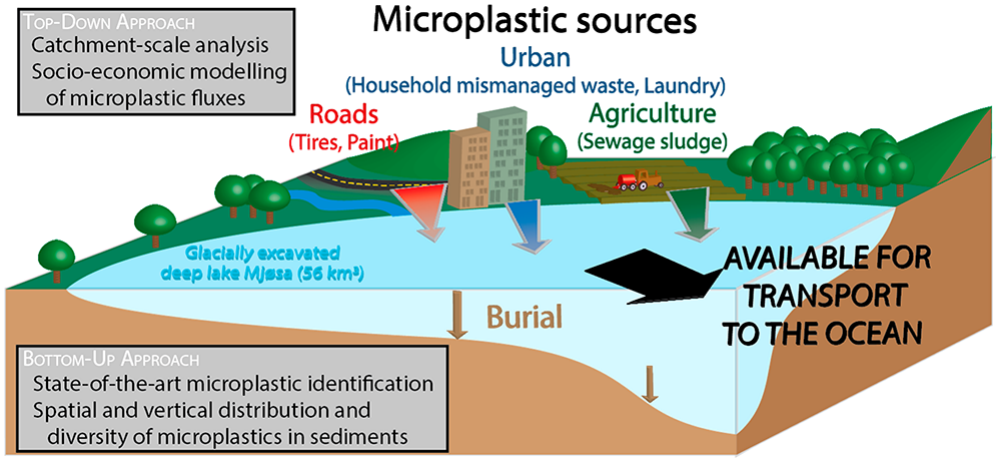

Microplastic research, initially focusing on marine environments, left freshwater ecosystems largely unexplored. Freshwaters are also vulnerable to microplastics and are likely the largest microplastic supplier to the ocean. However, microplastic sources, transport pathways, and fluxes at the catchment level remain to be quantified, compromising efficient actions toward mitigation and remediation. Here we show that 70–90% of microplastics reaching Norway’s largest lake, originating primarily from urban waste mismanagement and sludge application on crops, continue their journey toward the ocean without being buried. Indeed, our microplastic budget for the catchment shows that out of the 35.9 tons (7.4–119.4 t) of microplastics annually released into the lake, only 3.5 tons (1.3–8.8 t) are settling to the lake bottom. The spatial and vertical microplastic distribution and diversity in lake sediments, the socio-economic modeling of plastic fluxes and spatial information on land use and potential plastic sources all point toward urban and agricultural areas as emission hotspots of increasing importance. We conclude that the degree to which lake sediments represent a net microplastic sink is likely influenced by the nature of microplastics the lake receives, and ultimately on their origin.

## Abstract 2

Microplastics originating mainly from urban areas and sewage sludge application on crops bypass large Nordic lake and continue their journey to the ocean with limited burial.

## Introduction

Originally recognized as a problem in the marine environment, increasing amounts of microplastics are now also reported in terrestrial environmental samples around the world.^1^ Developing methods for microplastic source identification is a priority to enable actions toward mitigation and remediation. However, the sources of these items often lie on land where most of the plastic is used, rather than in marine and coastal ecosystems.^2^ Freshwater systems can act as transporters and/or sinks for terrestrial microplastics.^3^ Investigations into freshwater systems have been receiving increased attention in the past few years, although most of the focus is on reporting microplastic distribution.^4^ Hence, source tracking methods are currently limited^4^ and the factors influencing the behavior of freshwaters toward being transporters versus sinks are ill-known. Therefore, microplastic research efforts need to move upstream and improve catchment-level approaches to be able to identify the main sources and quantify freshwater microplastic fluxes and stocks.

Quantifying plastic release into surface waters is challenging due to data sparsity and difficulties attributing microplastics to their initial sources. Considering the standard waste mismanagement rate of 2%,^5^ and a release rate of 30%^6^ for mismanaged waste from coastal areas, we estimate that 106 tons of plastics could be annually released into Norway’s largest lake, Lake Mjøsa (5300 t of plastic waste annually processed in the catchment^7^). However, precise release pathways are influenced by land use with emission hotspots as highly populated areas^8^ and industrial and agricultural sites.^3,9^ In addition, the amount of microplastics deposited on the Lake bottom or that are transported downstream remain to be quantified.

Several studies investigated spatial variability in microplastic distribution in the Great Lakes and modeled the propagation of floating plastics.^6,10,11^ While these approaches are relevant for understanding plastic flows within large lakes, spatial information from the catchment were not included in these assessments limiting their usefulness for identifying the sources and release pathways.

The distribution of sediment contaminants can yield significant spatial and temporal information on pollution, although, in the case of plastics, a non-negligible fraction may not sink to the bottom due to their low density. Nevertheless, lake sediments provide reliable natural archives for estimating historical contamination of heavy metals^12−14^ and organic contaminants^15,16^ but their use to describe microplastics pollution has been limited so far.^17−19^

Here we present an original approach to investigate microplastic stocks and fluxes and apply it to a Nordic catchment-lake ecosystem, Lake Mjøsa. We quantify the different plastic morphologies identified along sediment cores collected at 20 sites and explain their spatial distribution with an innovative spatial data set. We report spatial information on land use, wastewater treatment plant (WWTP), urban and industrial services for the whole catchment as well as for delineated subcatchments where higher microplastic concentrations are reported. We further provide a first microplastic budget for the catchment where top-down estimates of microplastic emissions from the modeling of socio-economic activities are corroborated with sediment inventories.

## Materials and Methods

### Study Site

**Figure 1:**
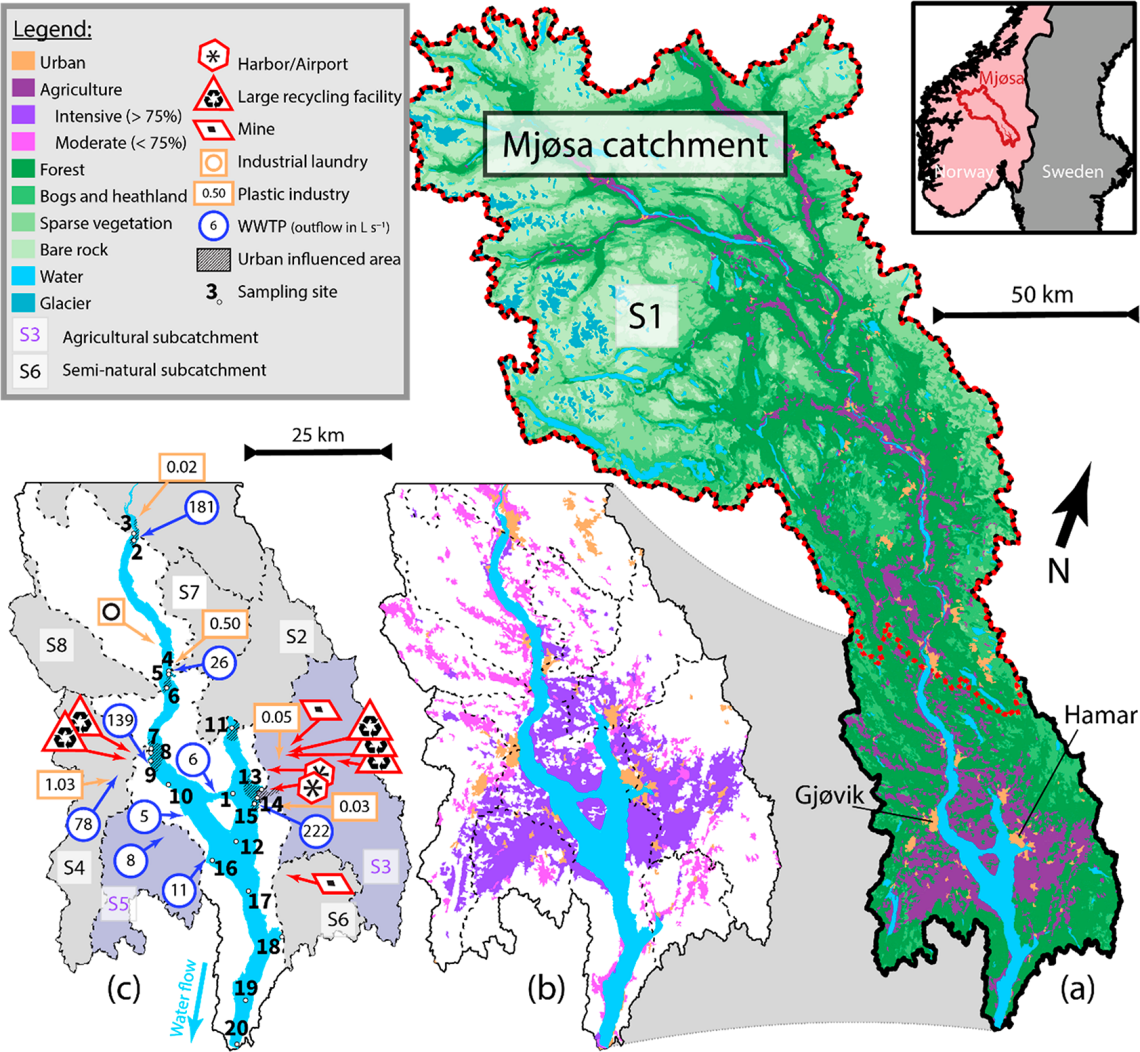
Land occupation, potential plastic emission sources, and sampling sites in the Mjøsa catchment and subcatchments. (a) Location map of the Mjøsa catchment and subcatchment 1. (b) Close up map of Lake Mjøsa showing subcatchment delineation, intensive and moderate agricultural areas, as well as urban areas. Note that agriculture, delineated by the purple color in panel a, is divided into two different land types and colors here. (c) Close up map of Lake Mjøsa showing sampling stations, agricultural and seminatural subcatchment delineation, and location of airport/harbor, mines, large recycling facilities, plastic industries, wastewater treatment plants (WWTP), and industrial laundries. Hatched areas are the high urban influence areas where sediment microplastic concentration is predicted using eq 1.

Mjøsa is a glacially excavated deep lake, the largest lake in Norway and one of the deepest in Europe with a volume of 56 km^3^ (Figure 1a; Table S1 of the Supporting Information, SI). Its catchment (17 028 km^2^) includes large mountain areas in the north, and forest, urban, and agricultural areas east and west of the lake. Most of the urban, industrial, and agricultural lands are located along the lake shoreline around the central part of the lake (Figure 1a). The river Gudbrandsdalslågen is the main tributary into Lake Mjøsa, draining an area of 13 000 km^2^, i.e., 78% of the catchment, with a higher proportion of natural land types than the areas around the lake (Figure 1a). Mjøsa is a drinking water source for approximately 70 000 people through the municipal and private water supply, as well as industry.

The 20 sampling sites within Lake Mjøsa (Figure 1c; Table S2) were chosen to be representative of a range of diverse lacustrine environments with some close to potential sources of microplastics, as well as reference points in Lake Mjøsa’s natural sediment accumulation areas. Several of the selected stations have been previously described regarding location, depth, sediment dating, and content of certain pollutants.^15^

Lake Mjøsa is subjected to internal sediment redistribution as a result of sediment focusing^20,21^ but also due to unstable sediment flow along steep underwater slopes (reaching locally 30°).^22^ Multibeam bathymetry and sub bottom profiler data are restricted to the surroundings of the city of Gjøvik^22^ (Figure 1a), which prevents any interpretation of the processes leading to sediment movements. To avoid misinterpretation of sediment microplastic concentrations, we only consider spatial and vertical patterns that are supported by several stations. To account for sediment focusing, however, we interpolated the sedimentation rate from four cores taken at different depth and dated with ^210^Pb for the 20 sampling sites^23^ (SI Note S1).

### Microplastics Sampling and Analyses

Sediment sampling was conducted between August 6^th^ and 9^th^, 2018. Cores were collected from each of the 20 locations using a Kajak-Brinkhurst sediment corer with an internal diameter of 8.5 cm. In the deepest parts of the lake a Van Veen grab was used and a core was taken once on the deck. Each sediment core was divided into 1 cm slices in the field, within 30 s for each core slice. On return to the laboratory, each core slice was freeze-dried for further processing by density separation with sodium iodide (NaI, 1.7 g cm^–3^), in some instances organic matter removal had to be included using Fenton’s reagent.^24^ Samples were passed through a 75 μm sieve to remove smaller particles. The retained material was then rinsed onto filter papers (GF/D, 47 mm, pore size 2.7 μm).

All particles found in each sample were analyzed by visual identification and measured along their longest and shortest dimension followed by chemical confirmation of the polymer material. Suspected microplastics were analyzed using a PerkinElmer Spotlight 400 μFT-IR in transmission mode. A diamond compression cell (DC-2 Diasqueeze) was used to improve spectral quality. Background scans were taken each time the compression cell was reloaded onto the instrument (circa every 1–4 suspected microplastic particles). All spectra were compared with a series of commercial (PerkinElmer ATR Polymers library, STJapan Polymers ATR library), the BASEMAN library^25,26^ and in-house libraries (including reference polymers, different textile materials, and potential sources of laboratory contamination) and manually inspected to confirm the match. Only 3% of the suspected microplastics were rejected after FT-IR analysis because of no polymer match. Procedural contamination was monitored throughout the sampling and analysis with the use of a series of blank samples to allow for results corrections based on presence of plastics in blanks. Only 1 out of 62 blanks contained one particle of pink polypropylene. Since this microplastic type was absent in our samples, no bank correction was performed. Data was presented as microplastics per gram dry weight for each core slice. The upper size class of microplastics included here is 5 mm. The lower limit of microplastics was defined by sample processing method (75 μm).

The diversity of microplastic polymers found in sediment samples was evaluated using the Shannon and Simpson diversity indexes, as well as richness and evenness indexes.^27−29^ We defined *S* as the richness, i.e., the total number of microplastic polymers in a sample, *n*_*i*_ as the number of microplastic of the *i*^th^ polymer, where *i* is an integer from 1 to S, *N* as the total number of microplastic in a sample (*N* = Σ_*i* = 1_^*S*^*n*_*i*_), *p*_*i*_ as the proportion of the i^th^ polymer () in a sample. The Shannon index *H* is defined as follows:
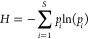
The Simpson index *D* is defined as follows:
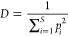
The evenness *E*_H_ is defined as follows:
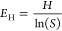
Mann–Whitney Rank Sum tests were performed to compare the diversity, evenness, and richness indexes reported for two sample groups. The Pearson and Spearman correlation coefficients were calculated to determine any significant linear relationship of these indexes with time.

### Sediment Age Model

The sediment age model developed with four dated sediment cores taken at various water depths^15^ provided an approximate date of deposition for each sediment layer at each station (see SI Note S1). As a result of sediment focusing,^20^ the sedimentation rate was higher for deeper sites although the variability in deposition dates was not significant compared to the duration of the studied time period. For clarity, we use only deposition dates averaged for all sites in our assessment. To look for spatially consistent temporal patterns rather than isolated signals, we calculated indexes with samples from a given sediment depth interval for a group of sites (all, urban or natural, as defined below).

### Catchment and Subcatchments Delineation

Mjøsa catchment and selected subcatchments (Figure 1) have been delimited using a 10 × 10 m^2^ Digital Elevation Model publicly available from hoydedata.no which was reclassified to 25 × 25 m^2^ for computational efficiency. The reclassified DEM was then processed with the TauDEM package^30^ in order to fill depressions and then obtain the D8 flow direction raster (all water from one cell flows to the lowest adjacent one). The NVE river network^31^ was burned into the DEM with filled depressions. The consolidated DEM was then used to derive the catchments which were further used in a GIS application (QGIS v.3.6.0) to retrieve land cover data.

### Land Cover Data

Land use data has been retrieved from a Jupyterhub intersecting the catchment and subcatchment polygons with the land use data from CORINE landcover from 2000 to 2018 (Copernicus European program of Earth observation). The various CORINE land classes have been reclassified as shown in Table S3. The total length of urban shoreline and the shortest distance from each sampling station to the nearest upstream town (>2000 inhabitants) was manually computed. Municipalities at least partly located in the catchment were identified; however, to construct a representative data set, only municipalities for which at least 50% of their territory was located within the catchment were included. The total surface area represented by the selected municipalities was only 7% larger than that of the catchment. Missing or added areas were mostly natural land types and all urban settlements with more than 2000 inhabitants were included. Official data on plastic industries, waste sorting facilities, waste production and management, population estimates, wastewater volume, driving distances and road length from the selected municipalities were then downloaded from Statistics Norway.^7^ The average water outflow was reported for each WWTP as provided in Snilsberg et al. (2005).^32^

Plastic industries were normalized according to the number of employees in the company, e.g., 1 and 0.5 normalized plastic industry are equivalent to a company of more than 100 employees and 50 to 99 employees, respectively. Plastic industries were then associated with the “Industrial and commercial unit” polygon found within the municipality boundary. In only one case, several industrial polygons were located within the municipality boundary. We therefore performed a web search for the main plastic industries in this municipality to confirm their location.

We identified five urban and industrial zones close to sites 2, 4, 7, 11, and 13 of which only three included plastic industries (close to sites 2, 4 and 13; Figure 1c). Subcatchments S3 and S5 are the most agricultural, with 28% and 45% agricultural areas, respectively. Most importantly, about 90% of their agricultural land is used for intensive agriculture (>75% of cultivation; Figure 1b). Some other subcatchments (S2, S4, S6, S7, and S8) also have a significant fraction of agricultural land (15 to 20%), but most of it falls into land types with <75% of cultivated land and considerable natural attributes. Other potential sources of microplastics, i.e., mines, airports, harbors, large recycling facilities, industrial laundry, and WWTP, represented in Figure 1c, also show a higher density close to sites 7 and 13, within the two largest urban centers, i.e., Gjøvik and Hamar.

### Top-Down Plastic Flux Estimates

The plastic fluxes for the Mjøsa catchment were estimated following a top-down approach with statistics on socio-economic activities within the catchment similar to Boucher et al. (2019).^3^ This procedure included the estimation of: (i) the magnitude of different sources in the plastic consumption and production processes; (ii) the losses to the environment; and (iii) the releases into surface waters and Lake Mjøsa.

Annual national and municipal data on waste production and management, population estimates, wastewater volume, plastic industries, driving distances, and road lengths were retrieved from Statistics Norway.^7^ Other data sources included PlasticsEurope^33^ and scientific literature (Tables S4 and S5).

#### Step 1: Quantification of the Magnitude of Plastic Sources within the Catchment

The plastic life-cycle assessment included three phases: production (including primary production and plastic conversion), use, and end-of-life. The production of plastic was downscaled from European and national statistics on plastic production and sales taking into account the number of plastic producers and their size (i.e., number of employees) within the watershed. The total amount of plastics in use was estimated from French and Swiss estimates from Boucher et al. (2019)^3^ and population within the catchment, and usage by market was downscaled from data given by PlasticsEurope 2019.^33^ Plastic end-of-life treatment was obtained from official statistics on household plastic waste and treatment from municipalities within the watershed and on plastic waste by sector at the national level.

#### Step 2: Quantification of Environmental Losses

Environmental losses included all microplastics released into the various compartments of the environment. Note that at this stage, some plastics, e.g., from household waste mismanagement, are still macroplastics but show a high potential for microplastic generation.

##### Wastewater Treatment Plants

Losses during wastewater treatment were calculated from data on microplastics removal efficiency from several wastewater treatment plants in Norway, including one within the watershed.^34,35^ Since microplastics from household laundry represent a significant fraction of those contained in wastewater influents,^35^ microplastic losses from textile were estimated considering generic washing habits per household and standard share of synthetic clothes and shredding rate reported in the literature.^36^

##### Agriculture

Microplastic losses from agriculture included two main processes: (i) the weathering of mulching films and (ii) the application of sewage sludge. Most of microplastics originating from household and laundry, industrial processes (e.g., blasting and shredding of plastics), and from the decomposition of plastic surfaces (e.g., polymeric paints) will be conveyed to municipal effluents and mainly be collected by wastewater treatment plants. Existing wastewater treatment processes effectively removes most of the microplastics from water and retain them in the sludge.^37^ In Europe and North America about 50% of sewage sludge is used as a fertilizer in agriculture.^9^ In Norway, a total of 71 505 tons (dry weight) of sewage sludge has been applied to agriculture in 2018.^7^ Data on wastewater microplastic concentration and removal efficiency from several wastewater treatment plants in Norway, including one within the watershed,^34,35^ and wastewater volumes from municipalities within the catchment are used to estimate the amount of microplastic applied to agricultural soils. Additional estimates are derived from official municipal statistics on agricultural sludge application and typical microplastic content in sludge reported in the literature.^38^ Plastic losses from mulching were downscaled from national mulching surface area and typical rates of mulch application and mulch recovery reported in the literature.^3^

##### Construction

Microplastic losses from construction primarily occurs during the building phase^3^ assuming that once plastics have been integrated into a building, losses are negligible and that during the demolition phase, waste is properly managed. Expanded polystyrene (EPS) represents the main losses from construction sites,^3^ and is used for its expanding property for insulation and typically undergoes a polishing step which emits plastic dust.^39^ Losses were estimated using typical EPS usage in construction, plastics used for construction in the catchment and typical EPS loss rate reported in the literature.^3^

##### Plastic Industry

Losses in production were estimated from standard rates of losses during delivery of primary plastic within the watershed as a function of the total plastic produced in the watershed.

##### Car and Lorry Tires

Plastic emissions from tires have been calculated from emission rates estimated for Norway^40−42^ using official statistics on driving distances within the watershed for four categories of vehicles and standard synthetic rubber composition of tires.^43^

##### Road Marking

Road paint is a major source of synthetic polymers such as methyl methacrylate.^40,41^ Road marking losses were quantified using official statistics on road length within the watershed and downscaling the amount of paint annually applied on Norwegian roads^41^ considering that old paint was not gathered prior to new paint application.

##### Household Waste Mismanagement

Even if the Norwegian waste management system is among the most efficient in the world,^44^ involuntary spill and illegal littering is still a probable and important source of microplastics for the environment. Tons of plastic waste, mainly from private use, are recurrently collected on lake beaches in Norway, including along Lake Mjøsa.^45^ Jambeck et al. (2015)^5^ considered a standard rate of 2% of waste mismanagement for plastics in occidental countries. Here, we take this 2% rate as the most pessimistic rate for microplastic losses and consider a rate, 1 order of magnitude lower, as a more realistic estimate for microplastic losses from waste mismanagement.^3^ These rates are typically used for macroplastic losses, attributing them to microplastics comes with the assumption that the amount of plastic present in the environment is at steady state, i.e., that the amount of macroplastics losses equals microplastic generation through degradation. We applied these rates to annual amounts of household plastic waste generated within the watershed, i.e., mainly food packaging. While the underlying assumption that macroplastic stocks in the environment are at steady state (losses equal microplastic generation) cannot be supported by our data set, it helps taking into account microplastics generated from illegal littering, which would have been ignored otherwise.

This analysis excluded microplastics from cosmetics, recreational products, medical waste, toys, household appliances, and furniture. Most of these items are likely not the dominant sources of microplastics for the environment, although, Boucher et al. (2019)^3^ considered equestrian gear as a major source, as well as balloons and fishing gear. Altogether, we consider that the top-down environmental loss estimates are conservative given the nonexhaustive list of sources.

#### Step 3: Release Pathways to Surface Waters

Once in the environment, microplastics can reach surface waters through several pathways described in detail elsewhere.^3,46^

##### Mismanaged Waste

Leaching of mismanaged waste, e.g., resulting from littering or forgotten plastics in agriculture, to adjacent surface water has been previously estimated at 15–40%, with a mean value of 25%.^5,6^ We used these values for microplastic releases from agriculture, construction, the plastics industry, and household waste mismanagement. Note that a recent study showed that >99% of microplastics applied from biosolids were likely exported to the aquatic environment.^47^ Given that, to our knowledge, only one study has specifically looked at microplastic agricultural runoff, and they argue that release rates are likely influenced by local properties and weather events,^47^ we used the mismanaged waste release rate for agriculture microplastic releases, although it is likely conservative.

##### Road Runoff

Microplastic emissions from tire dust and road paint are mainly released to surface waters through road runoff. Previous studies have estimated that 2–18% of these particles reached the surface waters.^40,48^ We considered a mean value of 6%.^3^

##### Sewage System

We used reported microplastic capture rates for wastewater treatment plant in Norway including one in the watershed.^34,35^ Microplastic removal efficiency was reported to be as high as 90 to 99% for these WWTP with a mean value of 95%. This release pathway is likely to provide a conservative estimate since we neglected any microplastic release caused by storm overflow.

##### Direct Release

This pathway was only applied to WWTP effluents, as microplastic capture rates were already applied during the calculation of the associated environmental losses (step 2).

### Microplastic Stock in Lake Mjøsa Sediments

To estimate the total mass of microplastic in the top 5 cm of the sediment column in L. Mjøsa, two cases have been considered: (i) sediment located within 2 km downstream of urban areas, defined as high urban influence areas; and (ii) sediment located in the rest of the lake bottom defined as natural areas. Mean sediment microplastic concentration ([MP]) in high urban influence areas has been modeled with the following equation:
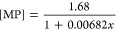
1where *x* (in m) is the distance to the nearest upstream town.

For the rest of the lake, i.e., for natural areas, given that mean sediment microplastic concentrations from the relevant stations were relatively consistent (e.g., ranging between 0.02 and 0.12 microplastic g^–1^), the mean, minimum, and maximum concentrations obtained have been extrapolated. Note that, out of the 20 stations sampled in this study, nearly half of them were located in high urban influence areas, defined as “urban sites”, while the rest were “natural sites”. The surface area under high urban influence (37 km^2^) has been manually computed in QGIS using land cover data. As a minimum and maximum estimate for the high urban influence areas, we considered a total length of urban coastline from 6.5 to 13 km as estimated above. To simplify calculations, the high urban influence areas have been idealized as a trapeze whose width and surface area were 6.5–13 km and 37 km^2^, respectively. This procedure yielded the total mass of microplastic over the top 5 cm of the sediment column. Considering the sedimentation rates and the temporal trend in microplastic deposition, we also estimated microplastic deposition in 2016 in the whole lake (Table S6).

## Results and Discussion

### Microplastic Abundance, Composition, And Diversity

Microplastics (75–5000 μm) were found in each sampled sediment core, even in more remote locations (Table 1). Size distribution shows that 60% of plastic particles were less than 1 mm in size, 36% of plastics were between 1–5 mm in size, and 4% of plastics were greater than 5 mm in size (not considered as microplastics; Figure S1). The sediment microplastic concentration averaged over each core ranged between 0.01 and 1.46 microplastic g^–1^ which is consistent with typical concentrations reported from freshwater sediments.^49,50^ Half of the selected sites, located in urban (and/or agriculturally) influenced areas (Table 1 and Figure 1c), showed mean concentrations above 0.20 microplastic g^–1^ and significantly higher concentrations than the other sites (Mann–Whitney rank sum test, *P* < 0.001). These sites are referred to as urban sites hereafter, while the remaining are so-called natural sites.

**Table 1:** Sediment Microplastic Concentration ([MP]) and Richness (*S*), Diversity (*H* and *D*) and Evenness (*E*_H_) Indexes at Each Sampling Site

sites		[MP] (microplastic g^–1^)						*S*	*H*	*D*	*E*_H_
		0–1 cm	1–2 cm	2–3 cm	3–4 cm	7–8 cm	average per site				
urban^a^	2	0.78	0.14	0.13	0.03	0.23	0.26	9	1.84	5.0	0.84
	4	2.54	0.36	0.85	0.10	0.04	0.78	6	1.46	3.5	0.82
	5	0.91	0.45	0.63		0.03	0.41	10	1.83	3.9	0.80
	7	1.18	1.44	0.20	0.38	0.18	0.68	11	2.05	6.0	0.85
	8	0.58	0.06	0.67	0.08		0.28	7	1.91	6.5	0.98
	9	0.93	0.07	0.29	0.09	0.03	0.28	8	1.80	4.6	0.86
	13	1.15	1.25	2.03	2.17	0.71	1.46	11	1.94	5.6	0.81
	14	0.75	0.93	0.92			0.52	6	1.39	3.0	0.78
	15	0.86	0.08	0.96	0.10		0.40	7	1.85	5.8	0.95
	16	0.20	0.37	0.18	0.41	0.06	0.25	8	1.87	5.7	0.90
natural	1	0.14		0.08	0.12		0.07	3	0.95	2.3	0.86
	3			0.04		0.15	0.04	4	1.33	3.6	0.96
	6	0.05		0.10		0.06	0.04	3	0.95	2.3	0.86
	10	0.24		0.20		0.07	0.10	3	1.05	2.8	0.96
	11^b^	0.13	0.24		0.24		0.12	4	1.24	3.0	0.90
	12	0.19					0.04	1	0.00	1.0	
	17	0.04					0.01	1	0.00	1.0	
	18	0.13					0.03	1	0.00	1.0	
	19		0.33	0.14	0.46		0.18	5	1.55	4.5	0.96
	20		0.05		0.15		0.04	3	1.04	2.7	0.95
average per depth	0.54	0.29	0.37	0.22	0.08						

a Sites showing average sediment microplastic concentrations greater than 0.20 microplastic g^–1^. All these sites, except one (site 16) which is located in an agriculture influenced area, are located in high urban influenced areas (Figure 1c).

b Site 11 is located near an urban zone (Figure 1c) but does not display patterns regarding microplastic sediment concentration, diversity, evenness, and richness that are similar to the other urban-influenced sites. It was therefore considered as a natural site.

This finding is in line with recent freshwater studies also reporting higher microplastic concentrations closer to urban areas.^49,50^ Most of the cores show increasing concentrations with decreasing sediment depth although local trends may differ. Variability within the reported cores arise from differences from one layer to another. These differences can be caused by changes in plastic inputs to the lake, but they also likely reflect local depositional and erosional processes. Our data set and the absence of high-resolution bathymetric data does not enable us to resolve any of these processes. Hence, we thereafter focus on the microplastic concentrations averaged for each site and spatial and temporal patterns that are supported by several stations.

**Figure 2:**
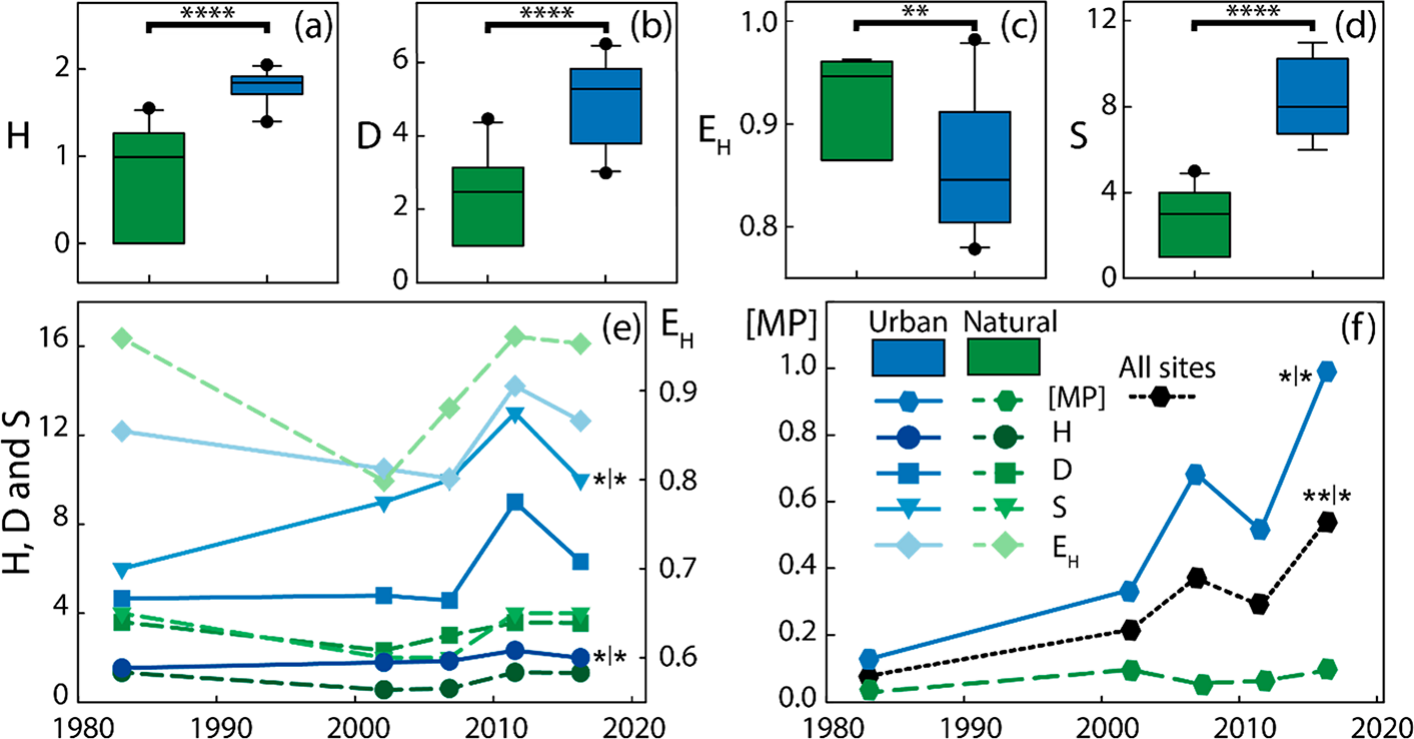
Sediment samples from urban sites showed higher microplastic concentrations and diversity indices than those from natural sites, as well as significant increasing trends with time. (a–d) Box plots of the diversity (*H* and *D* in panels a and b, respectively), evenness (*E*_H_ in panel c) and richness (*S* in panel d) indexes for sites located in natural and urban (and/or agricultural) influenced areas. (e) Temporal evolution of the diversity (*H* and *D*), evenness (*E*_H_) and richness (*S*) indexes. (f) Average sediment microplastic concentrations ([MP] microplastics g^–1^) for natural and urban (and/or agricultural) influenced sites. In panel (f), sediment concentrations are also shown for the sum of all sites. ****, ***, **, and * indicate significant difference following Mann–Whitney Rank Sum test (panels a–d), or significant positive relationship between a given index and time according to Pearson and Spearman correlation coefficients (panels e and f) at a level of 0.001, 0.01, 0.05, and 0.1, respectively.

A total of 13 polymers were found (Figure S2) with contrasting microplastic diversity across sites, but consistent patterns among urban and natural sites. Microplastic polymers were dominated by acrylic and polyester representing nearly 50%, followed by polyethylene, polypropylene, polyethylene terephthalate, polystyrene, viscose, and polyamide, while synthetic rubber, poly methyl methacrylate, polyurethane, polyvinyl chloride, and polycarbonate were quasi absent (Figure S2). The polymer density of sediment microplastics ranged from 0.9 to 1.5 g cm^–3^ with an average of 1.15 g cm^–3^. Only two polymers were less dense than water: polyethylene and polypropylene. In total, fibers accounted for 50%, fragments 49% and beads 1%. The urban sites, showing higher mean concentrations (>0.25 microplastic g^–1^), also displayed higher richness (*S*, see Methods for a definition of the indexes) and diversity (*H* and *D*) indexes as well as lower evenness (*E*_H_) than the natural sites. Indeed, urban sites had *H*, *D*, and *S* indexes significantly higher (*P* < 0.001) than natural sites, while *E*_H_ indexes were significantly higher (*P* < 0.05) for natural sites (Figure 2a-d). All these indexes are consistent with urban sites being closer to the microplastic pollution source(s) compared to natural sites. Indeed, higher diversity and richness are expected closer to the source. In addition, higher evenness at more distal natural sites also supports this interpretation since these sites receive microplastics following transport and redistribution during storm events. These random-like transport processes have the effect of dispatching microplastic across space, homogenizing their spatial distribution.

### Temporal Trends in Sediment Microplastic Concentrations in Lake Mjøsa

Figure 2e,f displays the sediment microplastic diversity, richness, and evenness indexes for urban, natural, and all sites, as well as the sediment microplastic concentrations from the 1980s to 2017 for the three groups of sites. The Shannon diversity index, the richness and concentration significantly increased at the urban sites since the 1980s. Further, the concentration for all sites showed significant increasing trends, while no significant trends were associated with the natural sites. Hence, the increase in the accumulation rate of microplastic over time in the Mjøsa sediments (+ 1.2 ± 0.4%) seems to mainly be caused by increased deposition at the urban sites (+ 2.2 ± 0.7%).

### Urban Areas as the Main Sources of Microplastics

**Figure 3:**
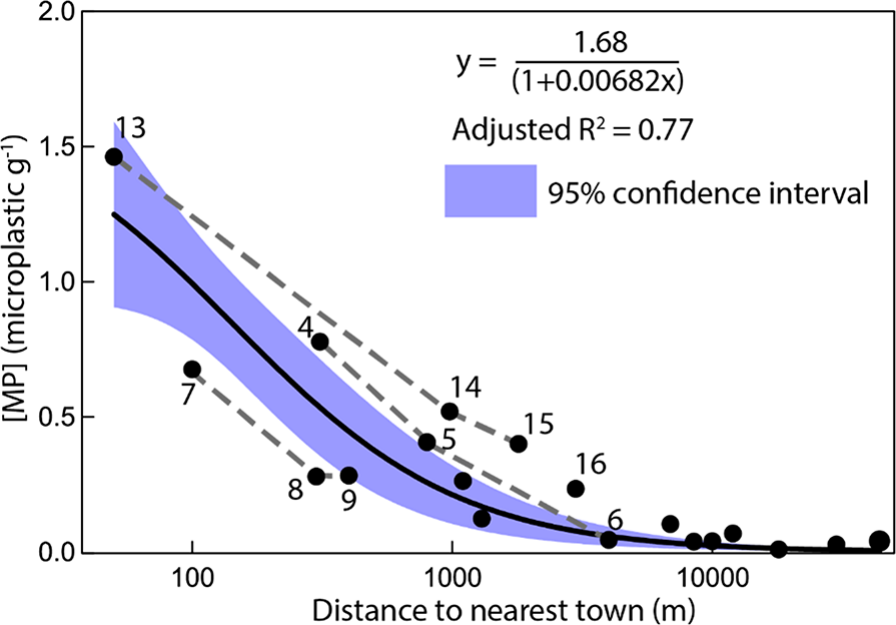
Sediment microplastic concentration ([MP]) shows a significant inverse correlation to the distance of the nearest upstream city. Depth-average [MP] at all sites as a function of distance to the upstream nearest town. Gray dashed lines connect stations along a littoral-distal transect, e.g., stations 13, 14, and 15. Note the systematic decrease in [MP] along these transects.

Figure 3 shows that there is a clear decreasing trend in sediment microplastic concentration as the distance from the nearest town increases. This observation is also supported by the three littoral-distal transects (stations 4–5–6, 7–8–9, and 13–14–15) showing a clear decreasing trend. The variability in sediment microplastic concentration ([MP] in microplastic g^–1^) can be well predicted (*R*^2^ = 0.77) using a simple inverse regression (eq 1). 

This finding points toward near-shore towns acting as the main source of microplastics to Lake Mjøsa which is consistent with similar findings in coastal ecosystems^51−54^ and the current view that a significant fraction of microplastics comes from urban waste mismanagement.^3,5,6,8,55,56^ Sediments collected at urban sites have a microplastic content with higher diversity and richness, as well as lower evenness which are all consistent with this interpretation. Similarly, richness and diversity increase with time at urban sites, but not at natural sites, suggesting that microplastic releases increase mainly from urban areas.

### Modeled Plastics Environmental Losses and Releases into Surface Water

**Figure 4:**
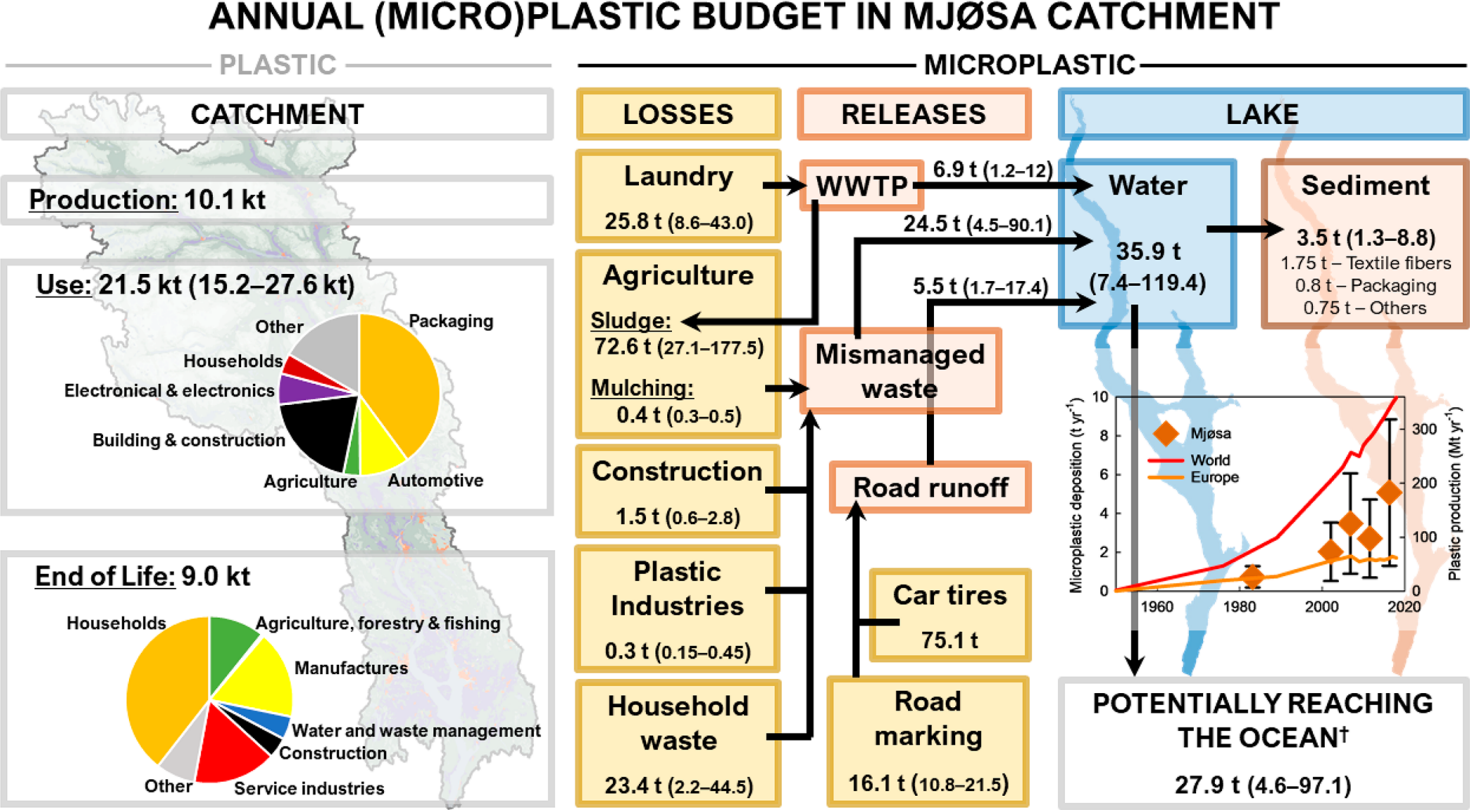
Annual (micro)plastic budget for Lake Mjøsa Catchment showing that most of incoming microplastics are not buried but that microplastic deposition increases exponentially following trends in Global plastic production. The magnitude of plastic sources within the catchment, environmental losses, and releases to the lake were calculated following a top-down modeling approach considering socio-economic activities within the catchment. Microplastic depositional rates show a significant fit to an exponential growth curve with a simple exponent and two parameters (Adjusted *R*^2^ = 0.87, *P* < 0.05). The doubling time of microplastic deposition in L. Mjøsa is about 12 years. Microplastic deposition is significantly correlated to world plastic production (Pearson’s correlation, *r* = 0.92, *P* < 0.05) and population in the catchment (Pearson’s correlation, *r* = 0.90, *P* < 0.05). World and european plastic production numbers are from PlasticsEurope^33,39^ while population date are from Statistics Norway.^7^ Microplastics from tires (4.5 t) were omitted from the microplastic flux potentially reaching the ocean because they are considered to be trapped in road verges, sedimentation ponds, or very littoral sediments.^60,61^

Our socio-economic top-down modeling reveals that about 10.1 kt of plastic are annually introduced into the market in Mjøsa catchment, while between 15.2 and 27.6 kt are estimated to be currently in use and only 9.0 kt discarded (Figure 4; Table S4). The imbalance between production and end-of-life fluxes can be explained by the increasing use of plastics as well as their increasing lifetime, or by unaccounted export fluxes.

Total microplastic losses in the watershed are 308 (235–441) t yr^–1^ with microplastics from tire abrasion, agriculture (mainly from wastewater sludge), laundry, household waste, and road markings as the main contributors (Figure 4; Table S5). Total releases into the catchment waters are 36 (7–119) t yr^–1^ with mismanaged waste being the main release pathway, mainly through the application of sludge on agricultural soils (16.3 t yr^–1^) and household waste littering (5.9 t yr^–1^). Releases from road runoff (5.5 t yr^–1^), including microplastic from tire and road paint, and from WWTP (3.8 t yr^–1^) represent about 20% and 12% of the total, respectively. Microplastic releases, totalling 160 g inhabitant^–1^ yr^–1^, are more than twice larger than those reported for Lake Geneva.^3^ However, about half of these releases are due to sludge application, a practice that has been banned in Switzerland since 2006.^57^

Release estimates from tires and road marking are robust since the tire weight before and after use, the amount of road paint that is applied and their release pathway are well constrained.^40,41^ Other fluxes, such as releases from sludge application on agricultural land or from household waste, have an intermediate level of uncertainty since initial stocks/fluxes are based on official municipal statistics but some losses and releases rates are taken from the literature, e.g., littering rate of 2%^5^ and 0.1%^3^ for household wastes. These uncertainties did not compromise the consistency of our budget since we used conservative release and loss rates. In particular, despite the use of a conservative release rate, both independent methods used to estimate microplastic releases from sludge application showed that this flux was the largest (Table S5).

Besides corroborating the importance of urban areas as microplastic sources, the top-down modeling highlights agricultural practices as potential considerable microplastic emission pathways as recently pointed out.^9^ Indeed, sludge application is the most important release within Mjøsa catchment (Figure 4) which is consistent with the fact that sites located at the outlet of most agricultural subcatchments, e.g., sites 13–16, show the highest sediment microplastic concentrations when normalized to the distance to the nearest town (Figure 3).

### Microplastic Lake Retention

Only about 10% of the microplastics emitted settles into Lake Mjøsa sediments (Figure 4), making the lake more of a pipe than a sink, in contrast to the interpretation for Lake Geneva.^3^ Our sediment microplastic stock estimate is likely conservative since size fractions <75 μm were not collected. Also, urban-influenced sediments showing higher sediment microplastic concentration might cover a larger proportion of the lake bottom than estimated here. However, this underestimation is likely not several fold ensuring that most of the plastics emitted in the catchment is not buried in Lake Mjøsa sediments. While the fate of those microplastics cannot be completely resolved, we can say with confidence that they are still available for further downstream transport or remobilization, potentially all the way to the ocean. The important contributions of sludge application (about 50%) and tire abrasion (about 15%) to microplastic releases in Mjøsa can partly explain the contrasted behavior of L. Mjøsa and Geneva. First, most of microplastics found in sludge from eight Norwegian WWTPs, including the largest one in Mjøsa catchment, are expected to float after remobilization since those are beads and fragments of low density (<1 g cm^–3^).^34^ In Switzerland, by contrast, sludge application was banned in the 2000s.^57^ Second, microplastics emitted from tires, e.g., Styrene Butadene Rubber,^42^ are associated with higher density components resulting in composite particles of very high density (1.5–2.2 g cm^–3^)^48,58,59^ that are expected to be trapped in road verges, sedimentation ponds or very littoral sediments.^60,61^ This interpretation is also consistent with the quasi-absence of synthetic rubber in our sediment samples (Figure S1). Furthermore, the dominant presence of high density (1.2–1.5 g cm^–3^) synthetic textile fibers in our sediment samples agrees well with synthetic textile fibers being the most abundant high-density microplastic in sludge.^34^ In contrast, fragments and films of polyethylene, polyethylene terephthalate or polyvinyl-chloride were the dominant microplastics found in Lake Geneva sediments.^62^

Microplastic deposition follows a similar exponential increase in Santa Barbara basin (doubling time of about 15 years)^63^ and Lake Mjøsa sediments (doubling time of ∼12 years; Figure 4), and shows significant correlations with increases in Global plastic production and local population in both cases^63^ (Figure 4). These relationships suggest a tight coupling between microplastic sedimentation and global production as well as household plastic usage and waste. This coupling is also highlighted in the microplastic budget for Mjøsa catchment where microplastics from municipal sewage sludge and from household mismanaged waste are the main environmental releases. However, the proportions of terrestrial microplastics being transported downstream or buried in freshwater sediments may differ from one catchment to another. Indeed, Lake Geneva was argued to represent a considerable sink for terrestrial microplastics,^3^ whereas Lake Mjøsa primarily acts as a transporter of microplastics which potentially reach the ocean (Figure 4). As discussed above, the microplastic emission pathways differ among both, Geneva and Mjøsa, catchments resulting in contrasted microplastic sedimentation processes. Hence, the proportion of terrestrial microplastics being trapped in freshwater sediments seems to be partly controlled by the nature of the local microplastics, and by extension, by their origin.

## Supporting Information Available

Supporting note on sediment dating; Figures S1 and S2 and Tables S1–S6 (PDF)
Microplastic data set (XLSX)
