# Supplementary material for: Bypass of Booming Inputs of Urban and Sludge-Derived Microplastics in a Large Nordic Lake
Source: Environ Sci Technol. 2021 Jun 1;55(12):7949–58. doi: 10.1021/acs.est.0c08443 (PMC8277126; doi:10.1021/acs.est.0c08443)
Supplement: Supplementary file 1 — es0c08443_si_001.pdf [file es0c08443_si_001.pdf]

Supplementary Information for
Bypass of booming inputs of urban and sludge-derived microplastics in a large Nordic lake

François Clayer[1*], Morten Jartun[1], Nina T. Buenaventura[1], Jose-Luis Guerrero[1] and Amy Lusher[1]

[1]Norwegian Institute for Water Research (NIVA), Gaustadalléen 21, 0349 Oslo, Norway
*Corresponding author: François Clayer
Email: francois.clayer@niva.no

This PDF file includes:

Supplementary Note 1 – Sediment dating
Figures S1 and S2
Tables S1 to S6
SI References

Supplementary Note 1 - Sediment dating:

Sediment cores collected from November 2005 to March 2006 from four stations at various water depths (50, 84, 261 and 440m) as reported by Fjeld et al. (2006)[1] were dated via gammaspectrometry at the Gamma Dating Center, Institute of Geography, University of Copenhagen in 2006.

Briefly, sediment cores were extruded with a Kayak-Brinkhurst sediment corer equipped with an acid-washed steel tube. The cores were sectioned at 1-cm intervals from the sediment surface down to 10-20 cm depth. The samples were transferred in field to annealed glass sealed with annealed aluminum foil and kept dark and cool at 4°C until analysis. At each station, 3-6 cores were taken and pooled together. Sediment samples were analysed for the activity of ^{210}Pb, ^{226}Ra and ^{137}Cs with a Canberra lsow-background Ge-detector. ^{210}Pb was measured via its gammapeak at 46,5 keV, ^{226}Ra via the granddaughter ^{214}Pb (peaks at 295 and 352 keV) and ^{137}Cs via its peak at 661 keV. The CRS-model has been applied[2].

All sediment cores showed a marked peak in ^{137}Cs which corresponds to the Chernobyl-accident in 1986 and was consistent with ^{210}Pb dating. Detailed descriptions of the cores are in Fjeld et al. (2006)[1].

All four cores showed a linear correlation between sediment depth and age (Fig. 1) within the top 8 cm of the sediment column. The slopes of the regression lines, i.e., the sedimentation rates (cm yr^{-1}) at each station, showed a linear correlation with water depth (Fig. 2). Increasing sedimentation rates with water depth are commonly observed in lacustrine sediments as the result of sediment focusing[3,4]. The regression equation shown in Fig. 2 has been used to estimate the sedimentation rate at each of the 20 sampling stations in this study and calculate the deposition date of each layer (Supplementary Table 2 below).

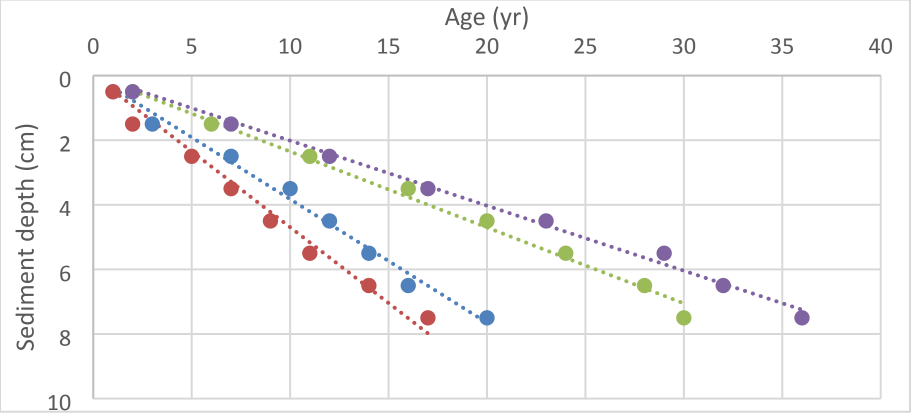

Figure 1: Sediment age as a function of depth for the four dated cores

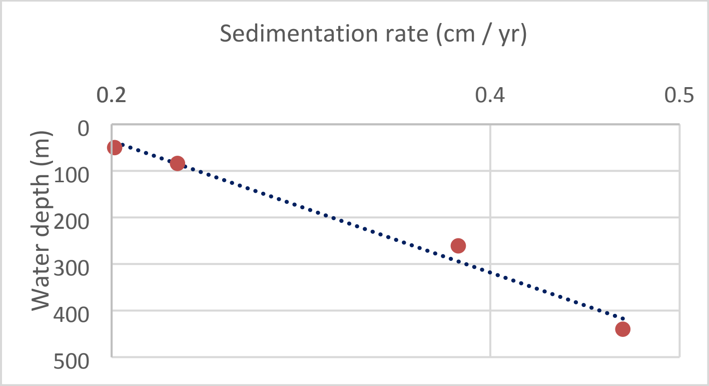

Figure 2: Sedimentation rate as a function of water depth.

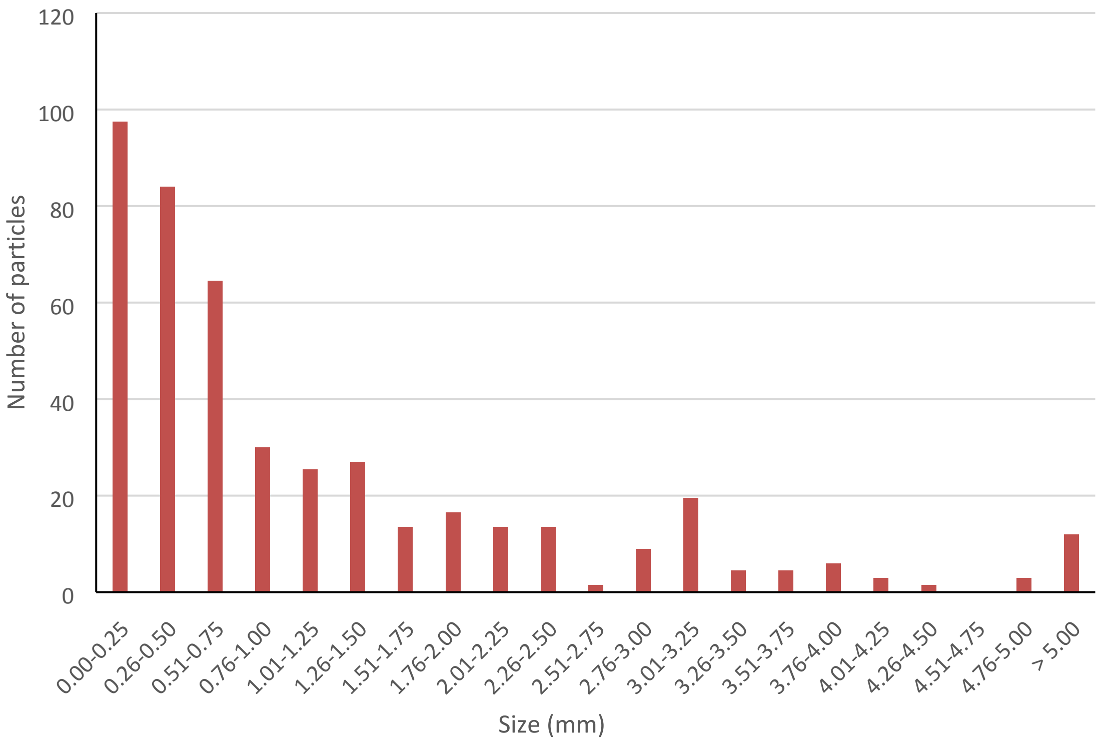

Figure S1: Size distribution of plastics extracted from sediments within Lake Mjøsa. There is an equal variance regarding size across cores.

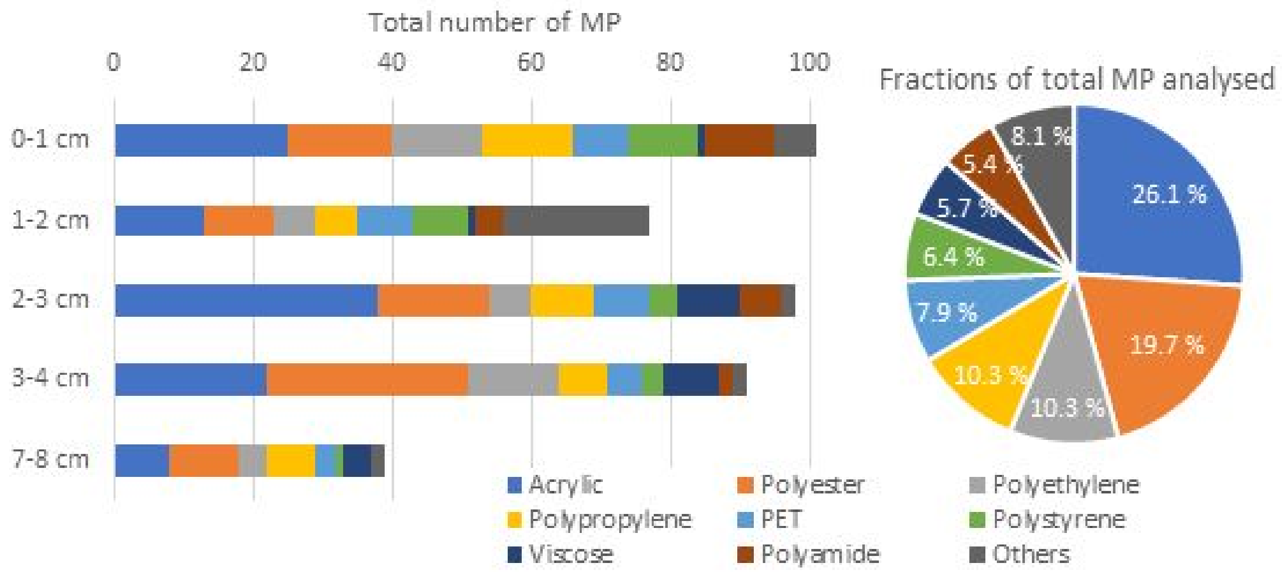

<u>Figure S2:</u> **Distribution of plastic polymers composing the microplastics found at all 20 sites within Lake Mjøsa displayed as a function of depth and as fraction of the total.** The most abundant polymers across all core depths were acrylic (26%) and polyester (incl. PET, 28%), while the less abundant polymers found were rubber, poly-methylmethacrylate, polyurethane, PVC and polycarbonate, each representing less than 3% (others). The average polymer density of sediment microplastics was 1.15 g cm^{-3}. Only two polymers were less dense than water: polyethylene and polypropylene. In total, fibers accounted for 50%, fragments 49% and beads 1%. Blue (41%) was the most prominent particle color found in Lake Mjøsa followed by red (21%) and green (17%).

<u>Supplementary Table 1:</u> Morphological and hydrological characteristics of Lake Mjøsa. Data from the Norwegian Water Resources and Energy Directory[5] and from Hobæk et al. (2012)[6]

Area	369 km^2
Mean depth	150 m
Max depth	453 m
Volume	55.361 km^3
Catchment	17 028 km^2
Average inflow from Gudbrandsdalslågen	256 m^3/s
Average runoff	321 m^3/s
Theoretical residence time	5.5 yrs

Table S2: Sampling stations coordinates, depth, distance to nearest upstream town and ages of its sediment layers.

Station ID	Lat. (° N)	Long. (° E)	Depth (m)	Distance to town (km)	Average date of sediment deposition				
					0 – 1 cm	1 – 2 cm	2 – 3 cm	3 – 4 cm	7 – 8 cm
**1**	60.771	10.9871	95	12.0	2016.6	2012.5	2008.3	2004.2	1987.7
**2**	61.0949	10.4513	30	1.1	2016.1	2011.0	2005.9	2000.8	1980.4
**3**	61.1037	10.4503	4	8.5	2015.9	2010.2	2004.6	1998.9	1976.4
**4**	60.9236	10.6797	5	0.31	2015.9	2010.2	2004.6	1999.0	1976.6
**5**	60.9208	10.6814	25	0.8	2016.1	2010.9	2005.7	2000.5	1979.7
**6**	60.8952	10.6897	54	4.0	2016.3	2011.6	2006.9	2002.2	1983.4
**7**	60.7986	10.6998	13	0.1	2015.9	2010.5	2005.1	1999.6	1977.9
**8**	60.793	10.7037	20	0.3	2016.0	2010.7	2005.4	2000.1	1978.9
**9**	60.779	10.7121	18	0.4	2016.0	2010.7	2005.3	2000.0	1978.6
**10**	60.752	10.7826	88	6.9	2016.6	2012.3	2008.1	2003.9	1987.0
**11**	60.8673	10.9272	43	1.3	2016.2	2011.4	2006.5	2001.6	1982.1
**12**	60.7024	11.0402	94	10.0	2016.6	2012.4	2008.3	2004.2	1987.6
**13**	60.7899	11.0691	8	0.05	2015.9	2010.3	2004.8	1999.2	1977.1
**14**	60.7756	11.0638	29	1.0	2016.1	2011.0	2005.9	2000.7	1980.2
**15**	60.7667	11.0653	29	1.8	2016.1	2011.0	2005.9	2000.7	1980.2
**16**	60.6638	10.9872	40	3.0	2016.2	2011.3	2006.3	2001.4	1981.7
**17**	60.6339	11.1142	450	18.0	2017.7	2015.6	2013.6	2011.6	2003.5
**18**	60.5831	11.2653	42	30.0	2016.2	2011.3	2006.4	2001.5	1982.0
**19**	60.4742	11.2139	317	37.0	2017.4	2014.9	2012.4	2009.9	1999.9
**20**	60.4059	11.2277	46	44.0	2016.3	2011.4	2006.6	2001.8	1982.5
**Avg (±σ)**			73 ±109	8.9 ±12.9	2016.3±0.5	2011.6±1.4	2006.8±2.3	2002.1±3.3	1983.2±7.0
**Urban (±σ)**			22 ±10	0.6 ±0.6	2016.0±0.1	2010.8±0.3	2005.5±0.5	2000.2±0.7	1979.1±1.5
**Natural (±σ)**			123 ±136	17.2 ±14.0	2016.6±0.5	2012.4±1.6	2008.2±2.7	2004.0±3.7	1987.2±8.0

Table S3: Land use classes

CORINE Land Cover Nomenclature	This study	
1.1 Urban fabric	Urban	
1.2 – 1.2.1 Industrial and commercial units		
– 1.2.2 Road and rail networks		
– 1.2.3 Port areas		
– 1.2.4 Airports		
1.3 – 1.3.1 Mineral extraction sites		
– 1.3.2 Dump sites		
– 1.3.3 Construction sites		
1.4 Artificial, non-agricultural vegetated sites		
2.1.1 Non-irrigated arable land	Agriculture	Intensive (> 75%)
2.3.1 Pastures		Moderate (< 75%)
2.4.2 Complex cultivation patterns		
2.4.3 Land principally occupied by agriculture, with significant areas of natural vegetation		
3.1 Forests	Forest	
3.2 Shrub and/or herbaceous vegetation associations	Bogs and heathland	
3.3 – 3.3.1 Beaches, dunes, sands	Sparsely vegetated areas	
– 3.3.2 Bare rock	Bare rock	
– 3.3.3 Sparsely vegetated areas	Sparse vegetation	
– 3.3.4 Glaciers and perpetual snow	Glacier	
5.1 Inland waters	Water	

Table S4: Magnitude of sources of plastic produced, used and discarded in Lake Mjøsa catchment

Type	Used parameters	Theoretical calculation	Calculation	Magnitude of Plastic sources
**Production**	Norwegian plastic sales [7]: 1.04 M€/yr<br>European plastic sales[8]: 360 M€/yr<br>European plastic production[8]: 61.8 Mt/yr<br>Normalized number of plastic industries† (Norway)[7]: 36.44<br>Normalized number of plastic industries† in the watershed[7]: 2.03	(National plastic sales) * (European plastic production) / (European plastic sales) / (# of plastic industries in Norway) * (# of plastic industries in the watershed)	1.04 * 61.8 / 360 / 36.44 * 2.06	10.1 kt
**Use**	Minimum:<br>Plastic use in France[9]: 4.6 Mt/yr<br>Population in France[9]: 67 M inhabitants<br>Maximum:<br>Plastic use in Switzerland[9]: 1.0 Mt/yr<br>Population in Switzerland[9]: 8 M inhabitants<br>Average:<br>Plastic use in Europe[8]: 51.2 Mt/yr<br>Population in Europe[10]: 527 M inhabitants<br><br>Population in the watershed[7]: 220788 inhabitants<br><br>Per domain[8]:<br>Packaging: 39.9% Building & Construction: 19.8%<br>Automotive: 9.9% Electrical and electronic: 6.2%<br>Agriculture: 3.4% Household and leisure: 4.1%<br>Others: 16.7%	Example for minimum estimate:<br><br>(Plastic use in France) / (Population in France) * (Population in the watershed)<br><br>Example for minimum estimate for Building & Construction:<br><br>(Plastic use in France) / (Population in France) * (Population in the watershed) * (% of plastic used in Building & Construction)	Example for minimum estimate:<br><br>(4 600 000) / (67 000 000) * (22788)<br><br>Example for minimum estimate for Building & Construction:<br><br>(4 600 000) / (67 000 000) * (22788) * (0.198)	Total plastic used in the watershed:<br><br>21.5 kt<br>Min: 15.2 kt<br>Max: 27.6 kt<br><br>Total plastic used for Building & Construction in the watershed:<br><br>4.26 kt<br>Min. 3.01 kt<br>Max. 5.46 kt
**End of life**	Plastic waste (from municipal waste treatment plants) from households within the watershed[7]: 3536 t/yr<br>National plastic waste by sector[7]:<br>Agriculture, forestry and fishing: 24 kt/yr<br>Mining and quarrying: 1 kt/yr<br>Manufacturing industries: 38 kt/yr<br>Electricity, gas, steam and air conditioning supply: 0 kt/yr<br>Water supply, sewerage, waste management and remediation activities: 10 kt/yr<br>Construction: 9 kt/yr<br>Service industries: 36 kt/yr<br>Other or unspecified: 17 kt/yr<br>Households: 88 kt/yr<br><br>Plastic waste treatment (from municipal waste treatment plants; for households waste only)[7]: recycling: 3512 t/yr; incineration: 23.4 t/yr.	Example for plastic waste from construction within the watershed:<br><br>(National plastic waste from construction) * (Plastic waste from households within the watershed) / (National plastic waste from households)	Example for plastic waste from construction within the watershed:<br><br>9 * 3536 / 88	Example for plastic waste from construction within the watershed:<br><br>362 t/yr<br><br>End of life – TOTAL:<br>9.0 kt/yr

† Plastic industries were normalized according the number of employees in the company, e.g., 1 and 0.5 normalized plastic industry are equivalent to a company of more than 100 employees and 50 to 99 employees, respectively.

Table S5: Magnitude of plastic environmental losses and releases into surface waters within Lake Mjøsa catchment

Type	Used parameters	Environmental losses					Releases into surface waters			
		Theoretical calculation	Calculation	Mean Value	Low value	High value	Calculation route	Mean Value	Low value	High value
**Agriculture**	Total agricultural area (Norway)[7]: 986 200 ha<br>Agricultural area with mulching (Norway)[11]: 2800 ha<br>Total agricultural area in the watershed‡: 158 075 ha<br>Fraction of mulch membranes forgotten onsite[9]: 0.03-0.05<br>Density of much films (LDPE): 0.9<br>Rate of application of mulch: 10%<br>Thickness of mulch films: 25 x 10^{-6} m<br><br>Sludge applied on agricultural soils in the watershed[7]: 3000 t/yr<br>Microplastic content in sludge[12]: 0.9-3.4-5.9 % (w/w)<br><br>Volume of wastewater treated in the watershed[7]: 26.2 hm^3<br>Concentration of microplastic in wastewater[13,14]: 50000 MP/m^3<br>Microplastic removal efficiency[13]: 90-95-99%<br>Sludge application on agriculture[15]: 25-50%<br>Average volume of microplastic[§]: 0.07 mm^3<br>Average density of microplastic: 1.3 g cm^{-3}	Mulching:<br>(Agricultural area with mulching in Norway) / (Total agricultural area in Norway) * (Total agricultural area in the watershed) * (Fraction of Mulch membranes forgotten onsite) * (Rate of mulch application) * (density of mulch films) * (thickness of mulch films)<br><br>Sludge:<br>Option 1: (Sludge applied on agricultural soils in the watershed) * (microplastic content in sludge)<br><br>Option 2: (Vol. of wastewater treated in the watershed) * (microplastic concentration in wastewater) * (microplastic removal efficiency) * (Sludge application on agriculture) * (average volume of microplastic) * (average density of microplastic)	Mulching:<br>2 800 / 986 200 * 158 075 * 10 000 * (0.03 +0.05) / 2 * 0.1 * 0.9 * 25 * 10^{-6}<br><br>Sludge<br>Option 1: 3000 * 0.034<br><br>Option 2:<br>26 200 000 * 50 000 * 0.95 * (0.25 + 0.5) / 2 * 0.07 * 10^{-9} * 1.3	0.4 t<br><br>102 t<br><br>42.5 t<br><br>TOTAL:<br>72.6 t	0.3 t<br><br>27 t<br><br>26.8 t<br><br>TOTAL:<br>27.1 t	0.5 t<br><br>177 t<br><br>59.0 t<br><br>TOTAL:<br>177.5 t	Mismanaged waste[16,17]<br>(15-25-40%)	0.1 t<br><br>25.5 t<br><br>10.6 t<br><br>TOTAL:<br>18.1 t	0.05 t<br><br>4.0 t<br><br>4.0 t<br><br>TOTAL:<br>4.1 t	0.2 t<br><br>70.8 t<br><br>23.6 t<br><br>TOTAL:<br>71.0 t
**Wastewater**	Textile[†]:<br>Households in the watershed[7]: 103411<br>Yearly number of washing cycles per household[18]: 165<br>Average load per washing cycle[18]: 3.5 kg<br>Share of synthetic textiles[18]: 48%<br>Leaching rate from synthetic textile[19]: 300-900-1500 mg/kg<br><br>Total:<br>Volume of wastewater treated in the watershed[7]: 26.2 hm^3<br>Microplastic concentration in untreated wastewater[13,14]: 50000 MP/m^3<br>Average volume of microplastic[§]: 0.07 mm^3<br>Average density of microplastic: 1.3 g cm^{-3}	Textile:<br>(Households in the watershed) * (yearly number of washing cycles per household) * (average load per washing cycle) * (share oh synthetic textiles) * (leaching rate from synthetic textile)<br><br>Total:<br>(Vol. of wastewater treated in the watershed) * (microplastic concentration in wastewater) * (average volume of microplastic) * (average density of microplastic)	Textile:<br>103 411 * 165 * 0.0035 * 0.48 * 900 * 10^{-6}<br><br>Total:<br>26 200 000 * 50 000 * 0.05 * 0.07 * 10^{-9} * 1.3	Textile:<br>25.8 t<br><br>Total:<br>119.2 t	Textile:<br>8.6 t<br><br>Total:<br>119.2 t	Textile:<br>43.0 t<br><br>Total:<br>119.2 t	Sewage system[13,14]<br>(1-5-10%)	Textile:<br>1.3 t<br><br>Total:<br>6.0 t	Textile:<br>0.09 t<br><br>Total:<br>1.2 t	Textile:<br>4.3 t<br><br>Total:<br>11.9 t
**Constructio n**	EPS use in construction domain[20,21]: 5.8 – 15% of total plastics used in construction<br>EPS loss rate[9]: 3.4 g/kg<br>Plastic used for Building & Construction in the watershed: 3.01-4.26-5.46 kt	(Plastic used for Building & Construction in the watershed) * (EPS use in construction) * (EPS loss rate)	4 260 * (0.058 + 0.15) / 2 * 3.4 / 1000	1.5 t	0.6 t	2.8 t	Mismanaged waste[16,17]<br>(15-25-40%)	0.4 t	0.1 t	1.1 t
**Plastic industry**	Losses per delivered tonnes of plastic[9]: 0.015-0.045 kg/t<br>Amount of plastic produced in the watershed: 10.1 kt/yr	(Amount of plastic produced in the watershed) * (Losses per delivered tonnes of plastic)	10 100 * (0.015 + 0.045) / 2 /1000	0.3 t	0.15 t	0.45 t	Mismanaged waste[16,17]<br>(15-25-40%)	0.08 t	0.02 t	0.2 t

**Automobile tires**	Tire abrasion rate (Norway)[22,23]: Passenger cars 132 mg/km<br>Busses 360 mg/km<br>Small lorries 140 mg/km<br>Heavy lorries 712 mg/km<br>% of synthetic rubber in tires[19,24]:<br>Passenger cars / Small lorries 24%<br>Busses / Heavy lorries 11%<br>Traffic volume in the watershed[7]: Passenger cars 1.75×10^{9} km<br>Busses 1.10×10^{7} km<br>Small lorries 3.56×10^{8} km<br>Heavy lorries 1.10×10^{8} km	(Tire abrasion rate) * (% of synthetic rubber in tires) * (traffic volume in the watershed)	Passenger cars:<br>$132 * 10^{-9} * 0.27 * 1.75 * 10^{9}$<br>Busses:<br>$360 * 10^{-9} * 0.14 * 1.10 * 10^{7}$<br>Small lorries:<br>$140 * 10^{-9} * 0.27 * 3.56 * 10^{8}$<br>Heavy lorries:<br>$712 * 10^{-9} * 0.14 * 1.10 * 10^{8}$	55.4 t<br>0.45 t<br>12.0 t<br>7.26 t<br>TOTAL:<br>75.1 t			Road runoff[25,26]<br>(2-6-18%)	3.33 t<br>0.03 t<br>0.72 t<br>0.45 t<br>TOTAL:<br>4.5 t	1.11 t<br>0.01 t<br>0.24 t<br>0.15 t<br>TOTAL:<br>1.5 t	9.98 t<br>0.08 t<br>2.15 t<br>1.31 t<br>TOTAL:<br>13.5 t
**Road marking**	Total road length for Lorries in Norway[7]: 49429 km<br>Total road length for Lorries in the watershed[7]: 5915 km<br>Application of plastics through road marking in Norway[25]: 90-180 t/yr	(Application of plastics through road marking in Norway) / (Total road length for Lorries in Norway) * (Total road length for Lorries in the watershed)	(90 + 180) / 2 / 49 429 *5 915	16.1 t	10.8 t	21.5 t	Road runoff[25,26]<br>(2-6-18%)	1.0 t	0.2 t	3.9 t
**Household waste mismanagement**	Plastic waste (from municipal waste treatment plants) from households within the watershed[7]: 3536 t/yr<br>Fraction of mismanaged waste[9,16,17]: 0.001-0.02<br>Fraction of population living in urban areas in the watershed[7]: 0.63	(Plastic waste from households within the watershed) * (Fraction of mismanaged waste) * (Fraction of population living in urban areas in the watershed)	3536 * (0.001 + 0.02) / 2 * 0.63	23.4 t	2.2 t	44.5 t	Mismanaged waste[16,17]<br>(15-25-40%)	5.8 t	0.3 t	17.8 t
			**TOTAL**	**195.8 t**	**117.7 t**	**340.1 t**	**TOTAL**	**35.9 t**	**7.4 t**	**119.4 t**

‡ Computed by GIS analysis (QGIS)

† MP losses from textile will mainly end up in sludge (90-99%)

§ The average volume of microplastic has been computed idealizing microplastics as cylinders using their measured longest length and shortest width. While this procedure likely yields realistic volumes for fibres (about 50% of all microplastic found in Lake Mjøsa sediments), it probably yields a conservative estimate of the volume of fragments (49%) and beads (1%)

Table S6: Microplastic content in Lake Mjøsa sediments

	Used parameters	Sediment microplastic content				
		Theoretical calculation	Calculation	Mean Value	Low value	High value
**Over 0 to 5 cm depth**	Total length of urban shoreline†: 6.5-13 km<br>Lake area with high urban influence†: 37 km^2<br>Lake area with low urban influence†: 332 km^2<br><br>Microplastic sediment concentration for high urban-influence areas:<br>$\frac{1.68}{1+0.00692D}$ MP g^{-1}<br>*where D is the distance from shore in m (0 to 2000m)*<br>Microplastic sediment concentration for low urban-influence areas: 0.02-0.12 MP g^{-1}<br>Sediment depth: 5 cm<br>Sediment dry content for high urban-influence areas: 0.4 g cm^{-3}<br>Sediment dry content for low urban-influence areas: 0.25 g cm^{-3}<br>Average volume of microplastic[§]: 0.07 mm^3<br>Average density of microplastic based on measurements: 1.15 g cm^{-3}	High urban-influence areas:<br>(Lake area with high urban influence) * (microplastic sediment concentration as a function of D and urban coastline) * (sediment dry content) * (sediment depth) * (Average volume of microplastic) * (Average density of microplastic)<br><br>Low urban-influence areas:<br>(Lake area with low urban influence) * (microplastic sediment concentration) * (sediment dry content) * (sediment depth) * (Average volume of microplastic) * (Average density of microplastic)	High urban-influence areas:<br>$\int_{D\,=\,1m\,(coast)}^{2000m\,(max.\ urban\ influenced\ area)} \left[\frac{1.68}{1+0.00692D} \times \left(\frac{13000 + D\left(\frac{37000-2\times 13000}{2000}\right) + 130}{2} \right. \right.$ [illegible]<br>* 0.4 * * 5 * 10^4 *0.07 * 10^{-6} * 1.039<br><br>Low urban-influence areas:<br>332 * (0.02 + 0.12) / 2 *0.25 * 5 * 10^{10} *0.07 * 10^{-6} * 1.15	16 t<br><br>23 t<br><br>TOTAL:<br>39 t	15 t<br><br>7 t<br><br>TOTAL:<br>22 t	18 t<br><br>40 t<br><br>TOTAL:<br>58 t
**Annual microplastic deposition in 2016**	Age of sediment at 5 cm depth: 10-17.5-25 yrs<br>Average sediment microplastic concentration over 0-1 cm depth: 0.54 MP g^{-1}<br>Average sediment microplastic concentration over 0-5 cm depth: 0.35 MP g^{-1}	(Sediment microplastic content over 0 to 5 cm depth) / (Average sediment microplastic concentration over 0-5 cm depth) * (Average sediment MP concentration over 0-1 cm depth) / (Age of sediment at 5 cm depth)	39 / 0.35 * 0.54 / 17.5	3.5 t	1.3 t	8.8 t

† Computed by GIS analysis (QGIS)

§ The average volume of microplastic has been computed idealizing microplastics as cylinders using their measured longest length and shortest width. While this procedure likely yields realistic volumes for fibres (about 50% of all microplastic found in Lake Mjøsa sediments), it probably yields a conservative estimate of the volume of fragments (49%) and beads (1%)

Supplementary References

(1) Fjeld, E.; Rognerud, S.; Enge, E. K.; Borgen, A. R.; Dye, C. *Miljøgifter i sedimenter fra Mjøsa, 2005-2006*; 5313; NIVA, 2006.

(2) Appleby, P. G. Chronostratigraphic Techniques in Recent Sediments. In *Tracking Environmental Change Using Lake Sediments: Basin Analysis, Coring, and Chronological Techniques*; Last, W. M., Smol, J. P., Eds.; Developments in Paleoenvironmental Research; Springer Netherlands: Dordrecht, 2001; pp 171–203. https://doi.org/10.1007/0-306-47669-X_9.

(3) Engstrom, D. R.; Rose, N. L. A Whole-Basin, Mass-Balance Approach to Paleolimnology. *J Paleolimnol* **2013**, *49* (3), 333–347. https://doi.org/10.1007/s10933-012-9675-5.

(4) Blais, J. M.; Kalff, J. The Influence of Lake Morphometry on Sediment Focusing. *Limnology and Oceanography* **1995**, *40* (3), 582–588. https://doi.org/10.4319/lo.1995.40.3.0582.

(5) NVE. Norwegian Water Resources and Energy Directorate - River Network https://gis3.nve.no/link/?link=elvenett (accessed Aug 6, 2020).

(6) Hobæk, A.; Løvik, J. E.; Rohrlack, T.; Moe, S. J.; Grung, M.; Bennion, H.; Clarke, G.; Piliposyan, G. T. Eutrophication, Recovery and Temperature in Lake Mjøsa: Detecting Trends with Monitoring Data and Sediment Records. *Freshwater Biology* **2012**, *57* (10), 1998–2014. https://doi.org/10.1111/j.1365-2427.2012.02832.x.

(7) Statistics Norway https://www.ssb.no/ (accessed Jul 10, 2020).

(8) PlasticsEurope. *Plastics – the Facts 2019 An Analysis of European Plastics Production, Demand and Waste Data*; 2019.

(9) Boucher, J.; Faure, F.; Pompini, O.; Plummer, Z.; Wieser, O.; Felippe de Alencastro, L. (Micro) Plastic Fluxes and Stocks in Lake Geneva Basin. *TrAC Trends in Analytical Chemistry* **2019**, *112*, 66–74. https://doi.org/10.1016/j.trac.2018.11.037.

(10) Eurostat. European population by country https://ec.europa.eu/eurostat/tgm/table.do?tab=table&language=en&pcode=tps00001&tableSelection=1&footnotes=yes&labeling=labels&plugin=1 (accessed Aug 6, 2020).

(11) Scarascia-Mugnozza, G.; Sica, C.; Russo, G. PLASTIC MATERIALS IN EUROPEAN AGRICULTURE: ACTUAL USE AND PERSPECTIVES. *Journal of Agricultural Engineering* **2011**, *42* (3), 15–28. https://doi.org/10.4081/jae.2011.3.15.

(12) Magnusson, K.; Eliaeson, K.; Fråne, A.; Haikonen, K.; Olshammar, M.; Stadmark, J.; Hultén, J. *Swedish Sources and Pathways for Microplastics to the Marine Environment*; C183; IVL Svenska Miljöinstitutet, 2016.

(13) Magnusson, K. *Mikroskräp i Avloppsvatten Från Tre Norska Avloppsreningsverk*; NR C 71; IVL Svenska Miljöinstitutet, 2014.

(14) Lusher, A. L.; Hurley, R.; Vogelsang, C.; Nizzetto, L.; Olsen, M. *Mapping Microplastics in Sludge*; 7215; Norsk institutt for vannforskning, 2017.

(15) Nizzetto, L.; Futter, M.; Langaas, S. Are Agricultural Soils Dumps for Microplastics of Urban Origin? *Environ. Sci. Technol.* **2016**, *50* (20), 10777–10779. https://doi.org/10.1021/acs.est.6b04140.

(16) Jambeck, J. R.; Geyer, R.; Wilcox, C.; Siegler, T. R.; Perryman, M.; Andrady, A.; Narayan, R.; Law, K. L. Plastic Waste Inputs from Land into the Ocean. *Science* **2015**, *347* (6223), 768–771. https://doi.org/10.1126/science.1260352.

(17) Hoffman, M. J.; Hittinger, E. Inventory and Transport of Plastic Debris in the Laurentian Great Lakes. *Marine Pollution Bulletin* **2017**, *115* (1), 273–281. https://doi.org/10.1016/j.marpolbul.2016.11.061.

(18) Pakula, C.; Stamminger, R. Electricity and Water Consumption for Laundry Washing by Washing Machine Worldwide. *Energy Efficiency* **2010**, *3* (4), 365–382. https://doi.org/10.1007/s12053-009-9072-8.

(19) Lassen, C.; Hansen, S. F.; Magnusson, K.; Hartmann, N. B.; Jensen, P. R.; Nielsen, T. G.; Brinch, A. *Microplastics: Occurrence, Effects and Sources of Releases to the Environment in Denmark*; Danish Environmental Protection Agency, 2015.

(20) REDILO. *Erhebung Der Kunststoff Mengenstr€ome in Der Schweiz (Schwerpunkt Polyolefine)*; 2008; p Stoff-Strom-Atlas Kunststoffe Schweiz.

(21) PlasticsEurope. *Plastics – the Facts 2013 An Analysis of European Latest Plastics Production, Demand and Waste Data*; 2013.
(22) Sundt, P.; Schulze, P.-E.; Syversen, F. *Sources of Microplastic- Pollution to the Marine Environment*; M-321|2015; MEPEX, 2014; p MEPEX.
(23) Vogelsang, C.; Lusher, A. L.; Dadkhah, M. E.; Sundvor, I.; Umar, M.; Ranneklev, S. B.; Eidsvoll, D.; Meland, S. *Microplastics in Road Dust – Characteristics, Pathways and Measures*; 7361; Norsk institutt for vannforskning, 2019.
(24) Svoboda, J.; Vaclavik, V.; Dvorsky, T.; Klus, L.; Zajac, R. The Potential Utilization of the Rubber Material after Waste Tire Recycling. *IOP Conf. Ser.: Mater. Sci. Eng.* **2018**, *385*, 012057. https://doi.org/10.1088/1757-899X/385/1/012057.
(25) Kole, P. J.; Löhr, A. J.; Van Belleghem, F. G. A. J.; Ragas, A. M. J. Wear and Tear of Tyres: A Stealthy Source of Microplastics in the Environment. *Int J Environ Res Public Health* **2017**, *14* (10). https://doi.org/10.3390/ijerph14101265.
(26) Unice, K. M.; Weeber, M. P.; Abramson, M. M.; Reid, R. C. D.; van Gils, J. A. G.; Markus, A. A.; Vethaak, A. D.; Panko, J. M. Characterizing Export of Land-Based Microplastics to the Estuary - Part I: Application of Integrated Geospatial Microplastic Transport Models to Assess Tire and Road Wear Particles in the Seine Watershed. *Science of The Total Environment* **2019**, *646*, 1639–1649. https://doi.org/10.1016/j.scitotenv.2018.07.368.
